# Studies using IPS cells support a possible link between ZIKA and microcephaly

**DOI:** 10.1186/s13578-016-0096-4

**Published:** 2016-04-26

**Authors:** Jia Guo

**Affiliations:** National Center for Biodefense and Infectious Diseases, George Mason University, Manassas, VA USA

**Keywords:** Zika virus, Human neural progenitor cells, Microcephaly

## Abstract

There is a suspected link between Brazilian babies born with microcephaly and Zika virus (ZIKV) infection. However, little is know about the brain cell targets and the mechanisms that Zika virus may cause microcephaly. A recent report demonstrated that Zika virus infection increases cell death and dysregulates cell-cycle, resulting in attenuated human neural progenitor cells growth. This study fills a major gap and serves as an entry point to establish a mechanistic link between Zika infection and microcephaly.

The recent dramatic rise of infant microcephaly in Brazil has drawn globe attention [1]. There have been more than 4800 confirmed and suspected cases of babies born with small brains and serious neurological complications, such as Guillain-Barre syndrome [1]. Both the medical and scientific communities are searching for a cause for this sudden rise of the disease. Zika virus (ZIKV), a mosquito-borne flavivirus, has topped the suspect list largely because of the coincidence of ZIKV spread with the rise of the disease. In additions, evidence suggesting a direct link of Zika with infant microcephaly starts to emerge. For example, the viral RNA has been found in placenta, amniotic fluid and brain tissues of stillborn babies with microcephaly [2–4]. However, little is know about the brain cell targets and the mechanisms that Zika virus infection may cause microcephaly.

A recent study from Dr. Guo-li Ming and colleagues of Johns Hopkins School of Medicine has filled a major gap in ZIKV biology. This new research may serve as an entry point to establish a mechanistic link between ZIKV and microcephaly [5].

By using a laboratory protocol of differentiating induced pluripotent stem cells (iPSCs) into forebrain-specific human neural progenitor cells (hNPCs) [6], Ming and co-authors identified that ZIKV efficiently infects hNPCs cells. Strikingly, they found that infected hNPCs can further release infectious ZIKV particles, supporting a spreading infection that can lead up to 90 % of cells being ZIKV positive. This new evidence supports that ZIKV could potentially infect and replicate robustly in neural progenitor cells, the very early cells that give rise to the bulk of the brain. Interestingly, ZIKV was found to be less infectious for more developed nerve cells in comparison with hNPCs. The result could suggest that fetal brains are likely much more susceptible to ZIKV than adult brains.

ZIKV infection was also found to lead to cell death by activating Cas3. The virus can dysregulate cell-cycle, resulting in attenuated hNPC growth. This is also consistent with the hypothesis that Zika infection may cause microcephaly.

This new study provides a tractable experiment system for modeling the impact of ZIKV on neural development. The in vitro hNPC model provides a new tool for investigating the underlying cellular and molecular mechanisms. The robust hNPC cellular phenotype comprises a readily scalable platform for high-throughout screens to anti-ZIKV drugs.
